# Diurnal adult resting sites and breeding habitats of phlebotomine sand flies in cutaneous leishmaniasis endemic areas of Kurunegala District, Sri Lanka

**DOI:** 10.1186/s13071-020-04154-7

**Published:** 2020-06-05

**Authors:** Tharaka Wijerathna, Nayana Gunathilaka

**Affiliations:** Department of Parasitology, Faculty of Medicine, University of Kelaniya, Ragama, Sri Lanka

**Keywords:** *Leishmaniasis*, Sand fly, Resting sites, Breeding sites, Sri Lanka

## Abstract

**Background:**

Sand flies are responsible for the transmission of several disease pathogens including *Leishmania*. Sand flies breed in habitats with high levels of humidity and organic matter. They are nocturnal in nature and peak activity ranges from dusk to dawn. The scientific evidence on breeding ecology and diurnal resting sites of sand fly fauna are important aspects of planning and implementing vector control activities. However, such fundamental information is grossly inadequate in Sri Lanka to support the control efforts in the country. Therefore, the present study addresses some of the important aspects of sand fly breeding ecology and diurnal resting sites.

**Methods:**

Potential resting sites were thoroughly observed, and sand flies were collected using a battery-operated aspirator and sticky papers when appropriate from three selected Medical Officer of Health (MOH) areas (Polpithigama, Maho and Galgamuwa) in Kurunegala district, Sri Lanka. Soil samples were collected from each potential breeding site. Half of each soil sample was incubated for 45 days. The other half was screened for immature stages. Adult sand flies collected from field and emerged adults at the insectary under confined incubation were identified using morphological characteristics.

**Results:**

Pepper bushes and termite mounds were the most notable resting sites while, betel bushes, cattle huts, piles of coconut shells, latrines, manna bushes and tree holes were also positive for sand fly adults. Only two species, *Phlebotomus argentipes* and *Sergentomyia punjabensis*, were reported. Soil samples were collected from a total of 432 sites and 7 of them were positive for immature stages. Predominant breeding habitats identified during the present study were mud flats and moist soils of rice paddies, the soil below decaying hay, drying irrigational tank bottom moist soil, and the floors of cattle huts.

**Conclusion:**

This study demonstrates that the potential adult resting sites and breeding habitats are abundant in the Polpithigama, Maho and Galgamuwa MOH areas. Therefore, vector control activities targeting both adult and immature stages of sand flies are recommended.
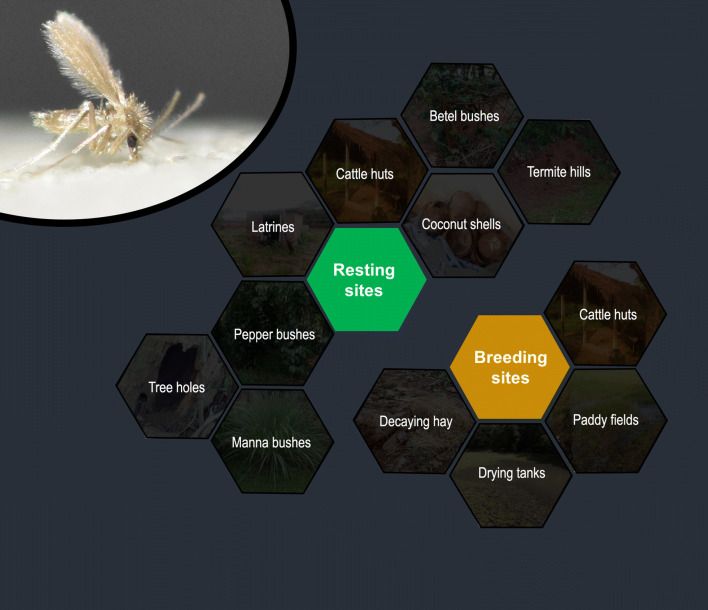

## Background

Phlebotomine sand flies act as vectors for several parasitic and viral diseases in the South Asian region [1, 2]. Of these, leishmaniasis transmitted by sand flies is considered as the most important and virulent infection. This disease has been recorded locally from Sri Lanka since 1992 with a few scattered cases until 2009 [3, 4]. In 2009, this was named as a notifiable disease [5]. Since then, patients have been reported from all over the country with a continuously increasing trend [6].

Sand flies are small crepuscular insects with peak activities lasting from dusk to dawn [7]. The stand flies may be a potential target of insect predators and subject to warmth at their resting places such as cattle huts, chicken sheds, vegetation, tree holes, and firewood stalks during the daytime [8–10]. People involved in outdoor activities around sand fly resting places during the daytime may increase their chance of exposing themselves to a bite from an infective sand fly. Therefore, information on sand fly resting places is useful in taking preventive measures against vector bites and planning of vector control activities.

Adult female sand flies lay eggs in the soil which provides a breeding environment for immature stages [11]. Certain *Phlebotomus* species such as *P*. *salengensis* and *P. sergenti* in subtropical areas in Asia prefer indoor places inhabited with both human and domestic animals for oviposition [9]. Caves are also a preferred habitat for some species including, *Sergentomyia bailyi*, *P. major* and *P. salengensis* [9]. In tropical forest areas such as Brazil, sand fly breeding mostly occurs in microhabitats associated with trees. Among these, the soil between buttress roots, tree bases and leaf litter are highly conducive for the breeding of certain species of the genus *Lutzomyia* [11]. However, under semi-arid and arid conditions, sand flies tend to select wall crevices in human dwellings, rodent burrows, and under stones as breeding habitats [12–14]. Bases of trees, tree buttresses and soil with cattle droppings are preferred by a range of different species irrespective of the geographical location [9, 11–14]. *Phlebotomus argentipes*, one of the commonest species in the South Asian region and known to be the vector for leishmaniasis transmission, prefers loose soil as a breeding ground [13]. A study from India emphasized that *P. argentipes* prefers indoor over outdoor habitats [15], while another study has reported that the immediate surrounding of houses is preferred as the breeding ground [16]. According to some studies, this species prefers cattle sheds over human dwellings [13, 17]. This uncertainty of breeding preference of sand flies warrants the importance of investigating the sand fly breeding ecology at the regional level.

Various larvicides and growth regulators are available for the control of sand flies [18]. Furthermore, new techniques such as the introduction of entomopathogenic bacteria and paratransgenesis are being developed to control *Leishmania* transmission [19, 20]. Improvements in these techniques beyond the laboratory largely depend on the understanding of sand fly breeding ecology.

A total of 20 sand fly species belonging to two genera have been reported from Sri Lanka [21]. However, no data are available regarding the breeding ecology and resting behaviour of sand flies in the country. The present study was conducted to identify the diurnal resting sites and larval breeding habitats of phlebotomine sand flies in three selected cutaneous leishmaniasis endemic areas in the Kurunegala district of Sri Lanka.

## Methods

### Study areas

**Fig. 1:**
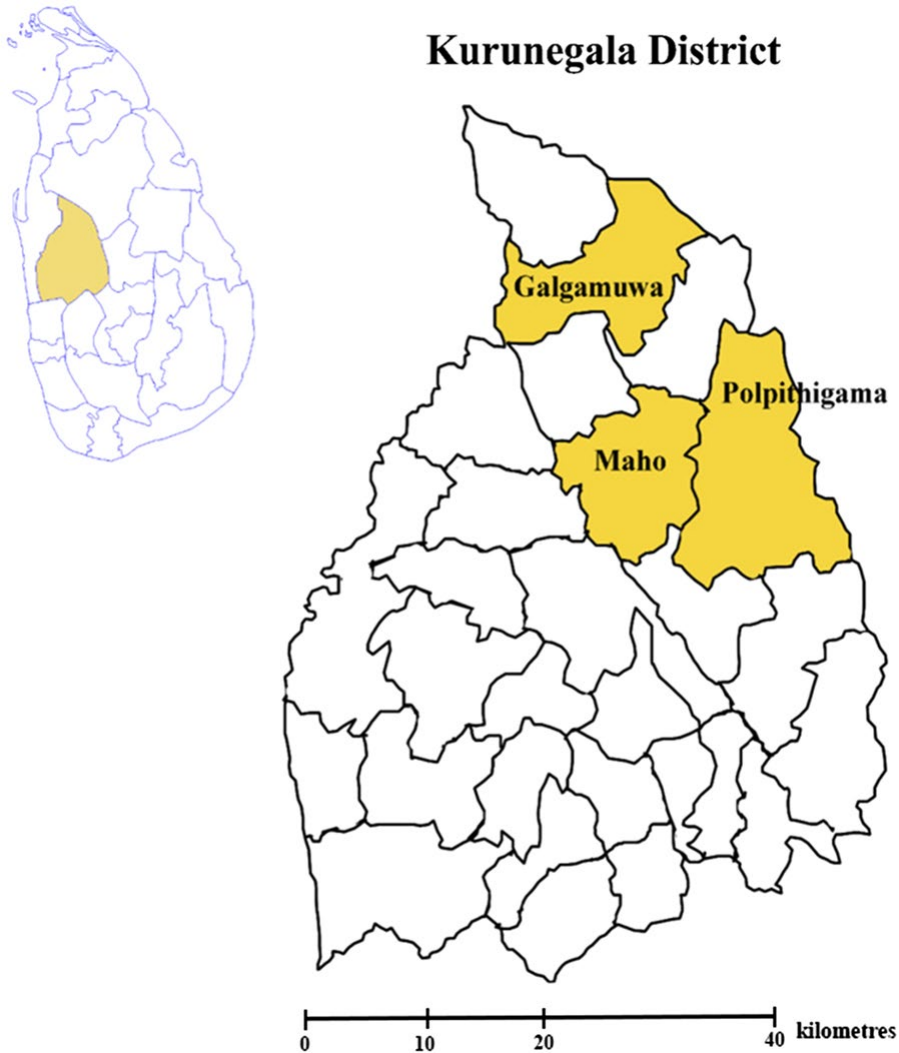
Map showing the three Medical Officer of Health areas where the study was conducted in Kurunegala district, Sri Lanka

The study was carried out at three Medical Officer of Health (MOH) areas namely; Polpithigama, Maho and Galgamuwa in the Kurunegala district (7°45′N, 80°15′E), North Western Province of Sri Lanka (Fig. 1). The selection of study sites was based on the higher prevalence of leishmaniasis patients during recent years (2009–2016). The Kurunegala district is situated approximately 116 m above sea level. It covers 4816 km^2^ of land area in the country with 1,610,299 inhabitants [22]. The district receives an average rainfall of 2095 mm annually. The average temperature and relative humidity are 31.7 °C and 69.6%, respectively [23]. The major activities of the population are agriculture and animal farming [22].

### Identification of potential breeding and diurnal resting sites

A preliminary survey was conducted during April 2017. Possible breeding and resting sites were identified. Checklists were prepared for the main breeding and resting site categories. Common sites such as man-made soil mounds, chicken pens (floor), pet shelters (floor), cattle huts (floor), wall crevices, abandoned sheds (floor), animal burrows, cracks in the soil surface, leaf litter, and termite hills were identified as breeding grounds for sand flies in previous studies [9, 11–14, 24], and were included in the checklist. These previously described sites are humid and rich in organic matter [11, 13, 14]. Therefore, sites with these basic characteristics were also included in the list though they are not commonly reported as breeding habitats of sand flies. These habitats include irrigational tanks, river-banks, rice paddies, soil below decaying hay, drying irrigational tank bottoms, pepper (*Piper nigrum*) and betel (*Piper betle*) cultivations.

The sites that have been reported ordinarily as diurnal resting places of sand flies were included in the resting site checklist based on previous data [8, 9, 13, 25]. These sites included animal burrows, buttress roots, cattle huts, chicken pens, dog shelters, latrines, leaf litter, tree holes, termite holes, fissures in rocks, crevices in walls, firewood stalks and bedrooms. Based on the common characteristics of these resting sites (dark and humid), other places with similar conditions were also included. These additional sites included banana bushes, coconut husks, brick piles, coconut shells, gliricidia (*Gliricidia sepium*) trees, manna (*Megathyrsus maximus*) bushes, pepper (*P. nigrum*) bushes and betel (*P. betle*) bushes.

### Inspection of potential diurnal resting sites and collection of adult sand flies

All encountered sites were examined for the presence of sand flies *via* an active visual search and aspiration method [13, 26]. The surveys were conducted monthly from May 2017 to December 2018, allocating three consecutive days (one day for each site). In each day, 5 h were spent conducting inspections (8:00–11:00 h and 13:00–15:00 h). One entomologist and a trained entomology field assistant were involved in the inspections and both were involved in observations to avoid the observer bias. Battery operated aspirators were used for collecting sand flies on visible surfaces. Sand flies that were in flight after the disturbance of the habitat were captured by swinging a sticky paper. The collected adult sand flies were placed in paper cups labeled with the location and type of habitat. The captured adult sand flies were transported to the insectary at the Department of Parasitology, University of Kelaniya, Ragama, Sri Lanka.

### Larval surveillance

Larval surveillance was conducted monthly from May 2017 to December 2018 along with the resting site inspections, in the same manner, allocating three consecutive days (one day for each site) for the search. In each day, 5 h were spent for searching and collection (8:00–11:00 h and 13:00–15:00 h). Potential breeding sites were inspected at each selected location. Emergence traps (60 × 120 cm and 60 × 60 cm depending on available space in the site) which were made of cloth nets (mesh size = 335 µm) employed to trap the emerging sand flies. The active visual search method was used to find the potential breeding sites in each MOH area. Soil/organic matter samples were also collected from each potential breeding habitat encountered using a spade and placed in 400 ml plastic containers. All containers with soil/organic matter were labeled with the breeding site category and date. Containers with soil/organic matter samples were maintained in conditions close to the standard rearing environment (26 ± 1 °C, 75–80% relative humidity), both during transport and in the insect rearing facility.

### Processing of soil samples

The soil samples were divided into two equal portions. The first portion was used to screen the immature stages through direct microscopic examination (magnification of 0.7–4.5×) and sugar flotation [27]. In the sugar flotation method, samples were suspended in a saturated sucrose solution and allowed to decant. The floating material was then examined for the presence of immature sand fly stages.

### Rearing of immature stages

The second portion of each soil sample in plastic containers was covered with a net cloth (mesh size = 335 µm). These containers were kept under standard larval and pupal rearing conditions (26 ± 1 °C temperature and 75–80% relative humidity). The containers were examined daily for 45 days to detect any emergence of adult sand flies. The soil in the containers was mixed by tapping against one hand while holding it with the other hand to facilitate the aeration twice a day, both in the morning and evening. At the end of a one and half month period, each soil sample was screened by sugar flotation for the presence of immature stages as described above.

### Species identification

Only the field-collected adult sand flies and those that emerged in the insectary from soil samples were identified to the species level. The adult sand flies were sacrificed by placing them in the freezer (− 20 °C). The specimens were placed in a 1.5 ml microcentrifuge tube using a fine paintbrush and/or fine forceps. Each specimen was soaked for 5 min in 70% alcohol, 90% alcohol and absolute alcohol, followed by xylene for dehydration. The dehydrated specimens were placed in lactophenol solution overnight to clear any remaining scales which makes the internal structures detectable [13]. The cleared samples were dissected removing the head and the terminal parts of the abdomen including spermatheca (in females) and gonostyles (in males). Specimens were mounted in Hoyer’s medium prepared in the laboratory and identified based on morphological characteristics using the available identification keys [13, 28, 29].

### Data analysis

The numbers of positive and negative sites between habitat categories were compared using the Chi-square test. The mean numbers of sand flies (or immatures) collected from different breeding and resting site categories were compared using the Kruskal-Wallis test.

## Results

### Diurnal resting habitats of adult sand flies

**Fig. 2:**
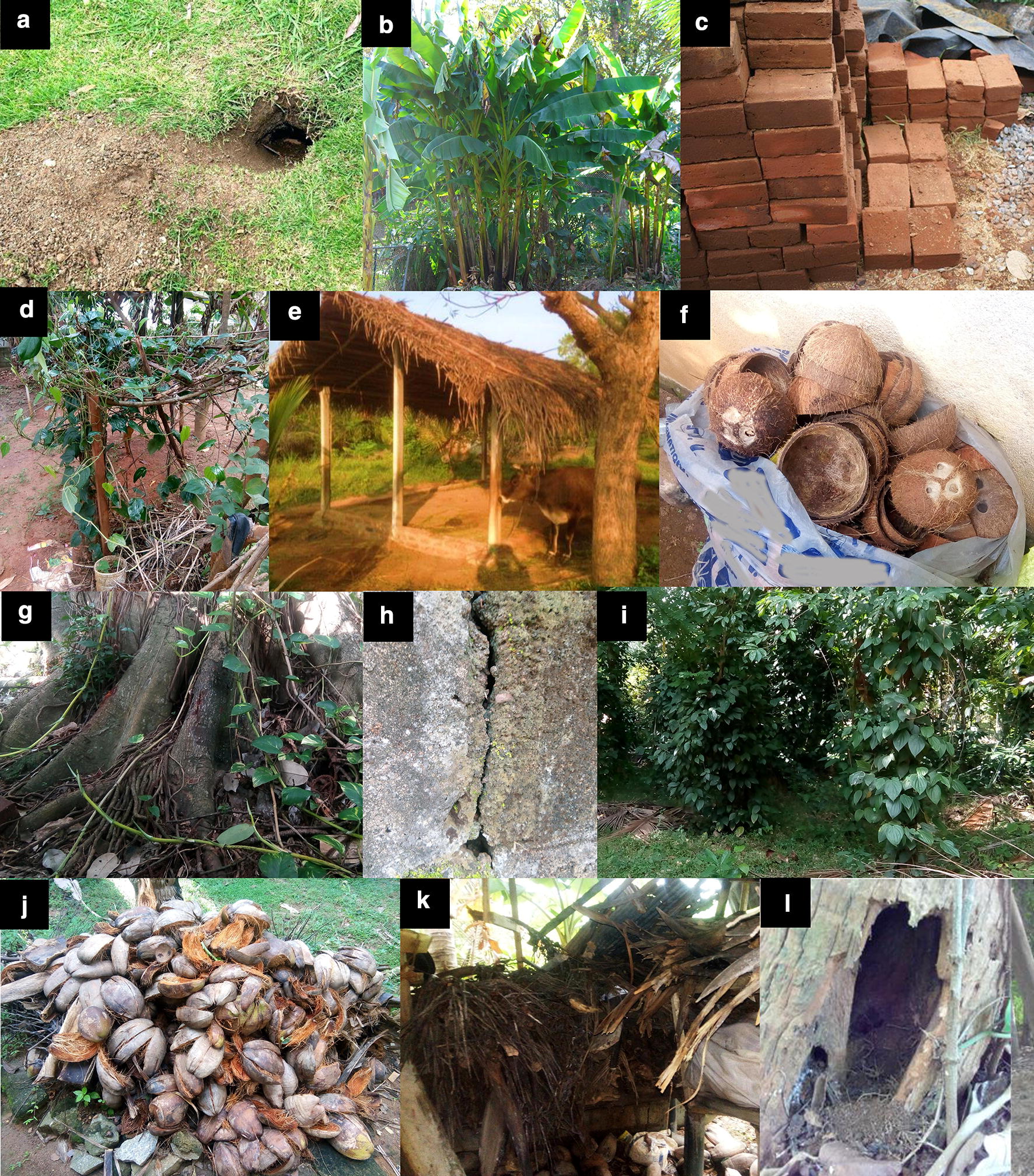
Resting and breeding sites of sand flies. **a** Animal burrow. **b** Banana bush. **c** Brick pile. **d** Betel bush. **e** Cattle hut. **f** Coconut shells. **g** Buttress root. **h** Wall crevice. **i** Pepper bush. **j** Coconut husk. **k** Firewood stalk. **l** Tree hole

A range of resting sites (*n* = 406) was examined during the study (Fig. 2). Sand flies were found in seven of these habitat types (Table 1). Only two species, *P. argentipes* and *S. punjabensis*, were identified. The most notable habitat categories were pepper bushes and termite mounds. Two of the observed sites from both categories were positive for adult sand flies. The known leishmaniasis vector in the South Asian region, *P. argentipes*, was the only species found from all the sites. *Sergentomyia punjabensis* was collected on a wall of a latrine. Other habitats such as cattle huts, piles of coconut shells, manna bushes, and tree holes were also found to be some of the preferred places where the sand fly adults seek protection as hiding places during the daytime. The number of positive and negative resting sites did not differ significantly across the different habitat types (*χ*^2^ = 8.000, *df* = 7, *P *>0.05) (Table 2). The number of individual sand flies per site was also not significantly different between the habitat categories (*P *>0.05) (Table 2).

**Table 1:** Potential diurnal resting sites of *P. argentipes* adults examined during the study

Resting site category	No. of sites sampled	No. of positive sites	Recorded species	No. of sand flies collected
Indoor habitats				
Latrine	28	1	*S. punjabensis*	1
Bedroom	15			
Crevices in wall	8			
Outdoor habitats				
Animal burrow	19			
Piles of coconut shells	6	1	*P. argentipes*	10
Brick pile	8			
Cattle hut	25	1	*P. argentipes*	7
Chicken pen	7			
Dog shelter	8			
Fissures in rock	6			
Fire wood stalk	28			
Piles of coconut husk	14			
Termite hole	43	2	*P. argentipes*	3
Vegetation associated habitats				
Banana bush	24			
Betel bush	6	1	*P. argentipes*	4
Buttress root	14			
Gliricidia plant	28			
Leaf litter	54			
Manna bush	15	1	*P. argentipes*	5
Pepper bush	18	2	*P. argentipes*	4
Tree hole	32	1	*P. argentipes*	2

**Table 2:** Comparative data for the number of positive and negative sites and the number of individuals per site for each resting and breeding habitat category

Habitat category	No. of positive sites	No. of negative sites	*χ*^2^	*P-*value	Mean no. of sand flies	*P*-value^a^
Resting sites						
Betel bush	1	5	8	> 0.05	4	> 0.05
Cattle hut	1	24			7	
Piles of coconut shells	1	5			10	
Latrine	1	27			1	
Manna bush	1	14			5	
Pepper bush	2	16			2	
Termite hole	2	41			1.5	
Tree hole	1	31			2	
Breeding sites						
Cattle hut (floor)	1	24	5	> 0.05	2	> 0.05
Rice paddy mud flat	3	11			2.67	
Rice paddy moist soil	3	8			4	
Soil below decaying hay	1	4			3	
Drying irrigational tank bottom moist soil	1	4			4	

^a^Kruskal-Wallis test

### Potential breeding habitats of sand flies

**Fig. 3:**
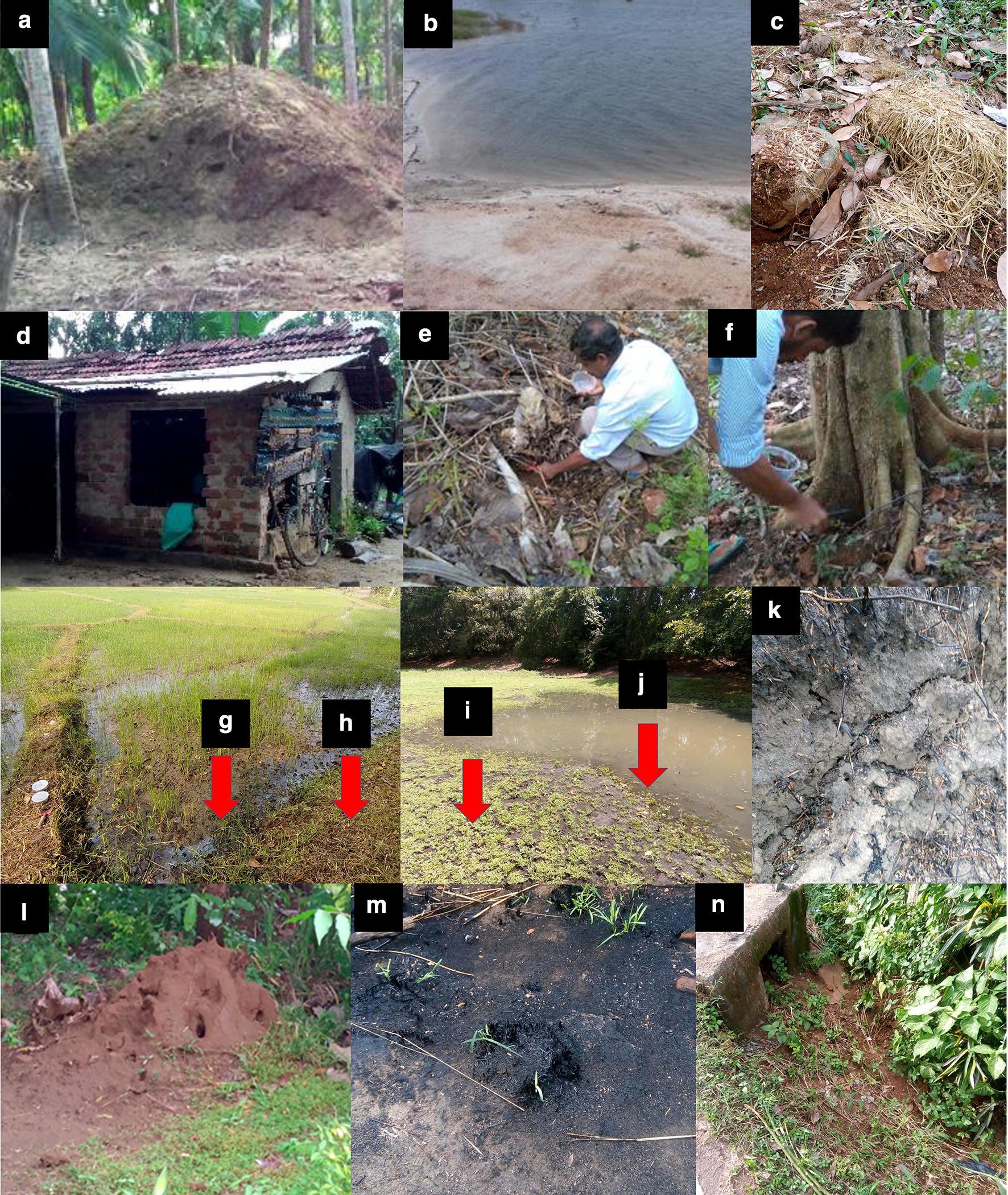
Potential breeding sites of sand flies. **a** Man-made soil mound. **b** Irrigational tank embankment. **c** Decaying hay. **d** Abandoned shed. **e** Leaf litter. **f** Soil between buttress roots. **g** Rice paddy mud flat. **h** Rice paddy moist soil. **i** Drying irrigational tank moist soil. **j** Drying irrigational tank mud flat. **k** Cracks on soil. **l** Termite mound. **m** Garbage burning site. **n** Drain

A total of 432 sites under 22 categories were identified as potential breeding sites (Table 3). Areas with leaf litter and man-made soil mounds were observed as the predominant habitats followed by rice paddy mudflats, rice paddy moist soil, cattle huts, and soil below decaying hay. Pupae were found in soil collected from rice paddy mudflats (Figs. 2, 3). Upon incubation under laboratory conditions, adult sand flies were retrieved from the moist soil collected from the bottom of a drying irrigational tank. All sand flies were identified as *P. argentipes.* No sand flies were collected from the emergence traps. The number of positive and negative sites did not differ significantly across different habitat categories (*χ*^2^ = 5.000, *df* = 4, *P *>0.05) (Table 2). On the other hand, the mean number of individual sand flies per site did not differ significantly between habitat categories (*P *>0.05) (Table 2).

**Table 3:** Potential breeding sites examined during the study and sites positive for sand fly immature stages

Breeding site category	No. of sites sampled	No. of positive sites (No. of sand flies encountered)			
		Larva	Pupa	Adult^a^	Total
Indoor					
Wall crevices	2				0
Abandoned shed (floor)	29				0
Outdoor					
Man-made soil mound	48				0
Chicken pen (floor)	7				0
Pet shelter (floor)	8				0
Cattle hut (floor)	25	1 (2)			1 (2)
Irrigational tank and river embankment	37				0
Animal burrow	19				0
Cracks in the soil surface	17				0
Drying irrigational tank bottom mud flat	10				0
Drying irrigational tank bottom moist soil	5			1 (4)	1 (4)
Termite hill	43				0
Garbage burning site	9				0
Drain	19				0
Vegetation associated					
Soil below decaying hay	5	1 (3)			1 (3)
Leaf litter	54				0
Rice paddy mud flat	14	2 (6)	1 (2)		3 (8)
Rice paddy moist soil	11	3 (12)			3 (12)
Pepper cultivation	18				0
Betel cultivation	6				0
Soil between buttress root	14				0
Tree hole	32				0

^a^After incubation

## Discussion

Information on basic biology, bionomics, ecology and environmental factors of disease vectors is fundamental evidence required for the implementation of control strategies. Knowledge on the resting behavior of adults and breeding habitats of vectors is crucial since all conventional vector control strategies target either elimination of adults or larval stages. Therefore, determination of the breeding habitats of sand flies along with their resting behavior is an essential pre-requisite when designing a leishmaniasis control programme. There may be considerable differences between different regions or geographical locations with diverse resting and breeding behavior of sand flies.

Phlebotomine sand flies are holometabolous insects with four life-cycle stages. Adult resting sites and immature breeding habitats of sand flies have been characterized by several studies in other countries [9, 14, 24, 25]. However, in Sri Lanka, available information on the above aspects are limited and grossly inadequate to design vector control programmes. To our knowledge, this study provides the first description of resting and breeding habitats of phlebotomine sand flies in leishmaniasis endemic areas of Sri Lanka.

The present study revealed that certain perennial crops, pepper and betel plantations, were the only resting places positive for adult sand flies, among all different agricultural areas investigated. These plants are vines grown around trees to make bushy structures creating shady and humid environments that provide favorable conditions for sand flies during daytime. It is interesting to note that some of the past patients recorded from these endemic areas were pepper cultivators. Therefore, these resting sites may provide good hiding places for adult sand flies and cultivators may become susceptible to infection through active bites of sand flies. However, more studies should be conducted to identify the exact reasons as to why the sand flies are associated with these microhabitats.

The selected areas in the present study experience extended drought periods throughout the year, with only one or two months of rain. Therefore, most of the microhabitats remain hot and dry throughout the year. Unlike in other resting places, sand flies were found in higher numbers in termite mounds at closer proximity to pepper and betel cultivations. Termite mounds provide moist and cool microenvironments despite the hot climate outside. This may be a reason why the sand flies have selected it as a diurnal resting place. According to some previous studies, other habitats such as crevices in walls, tree holes, firewood stalks, buttress roots, and animal burrows are considered as favourable resting habitats of adult sand flies [14]. However, during the present study, adult sand flies were not encountered from these habitats. This may be because these insects are small and well camouflaged, they are therefore difficult to find. However, the presence of these habitats in endemic areas cannot be disregarded since there is considerable potential in such sites to be used as resting places due to cool and humid conditions. Previous studies evidenced that *P. argentipes* in Sri Lanka is exophilic [30]. The present study also supports these findings, as no *P. argentipes* was found within houses. Night collections of sand flies during previous studies have indicated that *P. argentipes* were rarely found inside houses, further strengthening the argument of *P. argentipes* being exophilic in nature [31].

Most of the inhabitants in the areas studied depend on agriculture. Therefore, rice paddies are abundant around the villages. Rice paddies are rich in organic matter and nutrients that are continuously added as fertilizers for rice plants which creates a perfect microhabitat for the development of sand fly larvae. Although the evidence on the presence of sand fly larvae in rice paddies is scarce from other countries, the present study shows that rice paddies are preferred breeding habitats for immature sand fly stages in Sri Lanka.

The soil between buttress roots, tree holes, termite hills, and man-made soil mounds are known to be a common sand fly breeding ground, but we did not collect any sand flies from these habitats. As sand fly larvae are very small in size, detecting them in soil samples is highly laborious and requires continuous and thorough examination under the microscopes [13]; examiner errors are inevitable during such activities. Furthermore, one of the main assumptions that the investigators had to make was that the sand flies are evenly distributed (if present) in each of these habitat types. However, this is not necessarily correct in all cases. Although we used maximum precautions such as random sampling and replication to minimize these errors, one cannot directly exclude a potential breeding habitat due to the absence of sand fly immature stages from collected soil samples. Another important assumption is that all species have a similar chance of undergoing development in rearing cups. The efforts were taken to provide favourable conditions by aerating the samples twice a day, maintaining laboratory humidity and temperature at the optimum level for sand fly development.

In the sampling of adult sand flies, a direct visual observation approach was only used to collect resting sand flies by aspiration from the diurnal resting sites. On some occasions, where it was not possible to recognize flying insects as sand flies, sticky paper was used to capture the flying insects. Therefore, although aspiration resulted in most of the collections, a comparison of capturing efficiency of each technique may not be possible from these observations. Thus, the collection efficacy is not presented here since the main focus was to identify resting habitats by the presence of sand flies irrespective of the number captured. The use of the emergence trap was practically ineffective due to free-ranging cattle, monkeys and jungle fowl in these areas. Therefore, a site being positive by at least one method was considered in the study, without comparing the efficacy of the methods. However, studies which cover the aspects of species abundance, distribution and population dynamics and report on the collection efficiency among different techniques, would be useful to determine the appropriate collection methods that would be beneficial for control and surveillance programmes.

In the field-based application of advanced vector control strategies such as paratransgenesis and entomopathogenesis, the fundamental information on vector ecology and behaviour would be of paramount importance. Therefore, the knowledge generated through this study could be used to prioritize areas for intervention trials of the above novel vector control methods.

## Conclusions

Many different habitat types favorable for resting and breeding of phlebotomine sand flies are abundant in Polpithigama, Maho and Galgamuwa Medical Officer of Health areas in Kurunegala district, Sri Lanka. Pepper bushes, termite mounds and cattle huts were the resting sites positive for adult sand flies, while rice paddies, irrigational tanks, decaying hay, and cattle huts were identified as the breeding habitats positive for immature stages.

## Data Availability

Data supporting the conclusions of this article are included within the article. Raw data generated from this study will be available from corresponding author upon reasonable request.

## Abbreviation

MOH: medical officer of health
